# Postresuscitation care and prognostication after cardiac arrest—Does sex matter?

**DOI:** 10.1007/s00508-022-02026-x

**Published:** 2022-04-05

**Authors:** Julia Hasslacher, Hanno Ulmer, Georg Lehner, Sebastian Klein, Timo Mayerhoefer, Romuald Bellmann, Michael Joannidis

**Affiliations:** 1 Division of Intensive Care and Emergency Medicine, Department of Internal Medicine I, Medical University Innsbruck, Anichstr. 35, 6020 Innsbruck, Austria; 2 Department of Medical Statistics, Informatics and Health Economics, Medical University Innsbruck, Schöpfstr. 41/1, 6020 Innsbruck, Austria

**Keywords:** Biomarker, Mild therapeutic hypothermia, Neurological outcome, Pneumonia, Acute kidney injury

## Abstract

**Background:**

There are conflicting results concerning sex-specific differences in the post-cardiac arrest period. We investigated the sex distribution of patients after successful cardiopulmonary resuscitation (CPR), differences in treatment, complications, outcome and sex-specific performance of biomarkers for prognostication of neurological outcome.

**Methods:**

Prospective observational study including cardiac-arrest (CA) patients treated with mild therapeutic hypothermia (MTH) at 33 °C for 24 h or normothermia. We investigated common complications including pneumonia and acute kidney injury (AKI) and neuron-specific enolase, secretoneurin and tau protein as biomarkers of neurological outcome, which was assessed with the cerebral performance categories score at hospital discharge.

**Results:**

Out of 134 patients 26% were female. Women were significantly older (73 years, interquartile range (IQR) 56–79 years vs. 62 years, IQR 53–70 years; *p* = 0.038), whereas men showed a significantly higher rate of pneumonia (29% vs. 6%; *p* = 0.004) and a trend towards higher rates of AKI (62% vs. 45%; *p* = 0.091). Frequency of MTH treatment was not significantly different (48% vs. 31%; *p* = 0.081). Female sex was not associated with neurological outcome in multivariable analysis (*p* = 0.524). There was no significant interaction of sex with prognostication of neurological outcome at 24, 48 and 72 h after CPR. At the respective time intervals *p*_*interaction*_ for neuron-specific enolase was 0.524, 0.221 and 0.519, for secretoneurin 0.893, 0.573 and 0.545 and for tau protein 0.270, 0.635, and 0.110.

**Conclusion:**

The proportion of female patients was low. Women presented with higher age but had fewer complications during the post-CA period. Female sex was not associated with better neurological outcome. The performance of biomarkers is not affected by sex.

**Supplementary Information:**

The online version of this article (10.1007/s00508-022-02026-x) contains supplementary material, which is available to authorized users.

## Introduction

The average incidence of out-of-hospital cardiac arrest (OHCA) of presumed cardiac cause is high with 55 adults per 100,000 person-years [1]. Most of the patients are men [2–4]. Despite the fact that the incidence of OHCA in women is reported to be lower [5–8], its circumstances appear less favorable including higher age at arrest, a higher prevalence of non-shockable rhythms, fewer witnessed arrests, fewer arrests in public and less bystander cardiopulmonary resuscitation (CPR) [7–11].

In the post-resuscitation period, mild therapeutic hypothermia (MTH) seems to be underutilized in women [3, 12–15]. In the TTM2 trial women even exhibited a lower relative risk of death when treated with normothermia [16]. Furthermore, there are sex-specific differences in the adverse events profile during intensive care unit (ICU) stay. Whereas women are more likely to develop severe complications such as bleeding requiring transfusion and electrolyte disorders, men apparently show a higher incidence of pneumonia [17]. Furthermore, male sex may be associated with an increased incidence of acute kidney injury (AKI) requiring renal replacement therapy (RRT) [18]. Reported outcomes in women compared to men range from better [10, 19, 20] over similar [9, 21, 22] to worse [23, 24]. A similar variety of reports exists for neurological outcome [17, 23, 25–28]. Finally, little is known about the influence of sex on the performance of serum biomarkers for prognostication of neurological outcome; however, a variety of serum biomarkers have shown significantly different concentrations depending on sex and female hormone status possibly confounding diagnostic processes [29].

Consequently, it was the aim of our study to analyze the sex distribution of cardiac arrest (CA) patients admitted to ICU after successful CPR, differences in the post-CA period including treatment as well as adverse events and outcome. Furthermore, we wanted to investigate the sex-specific performance of biomarkers for prognostication of neurological outcome.

## Patients, material and methods

**Fig. 1:**
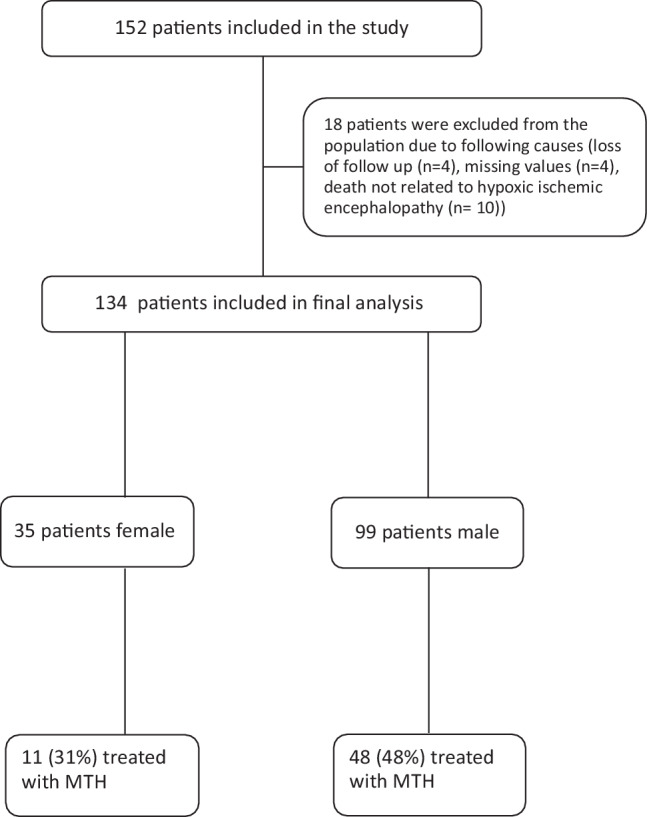
Flow chart for patient selection. *MTH* mild therapeutic hypothermia

This is a secondary retrospective analysis of a previous observational single center trial including 152 consecutive adult patients (age ≥ 18 years) with an in-hospital or OHCA with presumed cardiac cause admitted to the medical ICU of the University Hospital of Innsbruck from September 2008 to April 2013 after successful CPR [30]. The presence of a neuroendocrine tumor, stroke, intracranial hemorrhage or trauma as non-cardiogenic cause of CA as well as life expectancy of less than 24 h as determined by the treating physicians were considered as exclusion criteria. Furthermore, 18 patients were excluded from the cohort of 152 patients due to the following causes: loss of follow-up *n* = 4, missing values *n* = 4, death not related to hypoxic ischemic encephalopathy (*n* = 10), leaving 134 patients eligible for final analysis. (Fig. 1).

The CA data such as the rate of bystander resuscitation, time to return of spontaneous circulation (ROSC) and first monitored rhythm were collected from the emergency or heart alarm protocol according to the Utstein style [31]. An MTH was routinely applied to comatose patients with an initially shockable rhythm who had received advanced life support within 15 min and showed a ROSC within 60 min after collapse and in patients with non-shockable rhythms, if the event was observed and time to ROSC less than 25 min. Patients who underwent MTH were maintained at a target temperature of 32–34 °C for 24 h using an intravascular cooling device (Thermogard XP, Zoll Circulation, Incorporation, San Jose, Californnia, USA) and then gradually rewarmed at a rate of 0.2–0.4 °C/h. Furthermore, we assessed the total duration of hypothermia including cooling down, time on target temperature and rewarming (in hours).

To diagnose pneumonia, clinical and radiological criteria had to be fulfilled: clinical criteria were documented fever (≥ 38 °C) or hypothermia (< 35 °C), an abnormal total peripheral white cell count (> 10,000 or < 4500/µl), new-onset purulent sputum or change in the character of sputum, plus at least one of the following two features: auscultatory findings of rales on pulmonary examination, evidence of pulmonary consolidation, acute changes made in the ventilator support system to enhance oxygenation, as determined by arterial blood gas analysis or a worsening ratio of partial pressure of arterial oxygen to the fraction of inspired oxygen (PaO2/FIO2 < 240). Radiographic criteria were the presence of new or progressive and persistent infiltrate characteristic of bacterial pneumonia or a new consolidation in chest X‑ray on 2 following days.

The occurrence of AKI was determined according to the Kidney Disease Improving Global Outcomes (KDIGO) guidelines, based on serum creatinine values [32]. We also documented the patients who received renal replacement therapy (RRT). Patients were excluded from AKI staging if chronic kidney disease (> CKD G3) was described in the previous medical history and no baseline creatinine level was available.

### Biomarkers of neurological outcome

Blood samples were drawn from the arterial catheter at the day of CPR up to 7 days and assigned to following time intervals: 0–24 h (day 0), 24–48 h (day 1), 48–72 h (day 2), 72–96 h (day 3), 96–120 h (day 4), 120–144 h (day 5), 144–168 h (day 6). We determined serum neuron-specific enolase (NSE) as a gold standard for neuroprognostication [33], secretoneurin (SN) as early biomarker [30] and serum tau as a marker for axonal damage [34]. The SN measurements were performed with a radioimmunoassay according to Kirchmair et al. [35], NSE levels were measured with an electrochemoluminescence assay (ECLIA, Roche, Mannheim, Germany). Serum tau levels were determined with a commercial ELISA (Innogenetics/Fujirebio Gent, Belgium) detecting total tau as reported previously [36]. In retrospective analysis concerning biomarkers of neurological outcome, we focused on the first 72 h after CA.

Additionally, serum lactate (Roche, Mannheim, Germany), Sequential Organ Failure Assessment (SOFA) and Acute Physiology and Chronic Health Evaluation II (APACHE II) scores as well as the use of catecholamines were documented at the day of ICU admission. In all patients, we documented the ICU length of stay (LOS, in days). Patients were allocated to following subgroups: (1) withdrawal of therapy, removal of a therapy that has been started in an attempt to sustain life but is not, or is no longer, effective, (2) withholding of therapy, the decision not to make further therapeutic interventions, (3) maximal therapy. Subgroups (1) and (2) were defined as withdrawal of life-sustaining therapies (WLST). A detailed description is given in the Supplementary material.

To assess neurological outcome the cerebral performance categories (CPC) scale was used [37, 38]. To receive binary outcome parameters patients were classified into two groups according to their neurological outcomes: “good outcome” (CPC 1 and 2) and “poor outcome” (CPC 3–5). The CPC was assessed by a physician either before death at our ICU or in survivors at discharge from hospital (also including long-term acute care facilities) following a standardized protocol. The CPCs for patients transferred to another hospital were determined by follow-up telephone interview with the patient, family members or personnel of the rehabilitation facility at hospital discharge. A detailed description is shown in the Supplementary material.

Survival was determined at hospital discharge. For the standardized mortality ratio (SMR) we built the ratio between observed and expected deaths calculated from the APACHE II score.

The study protocol was approved by the Ethics Committee of the Medical University of Innsbruck (protocol number UN3493 272/4.31). Written informed consent was obtained from next of kin or retrospectively from patients who recovered.

### Statistical analysis

Categorical data are given as counts and percentages, continuous data as medians and interquartile ranges. Normal distribution of continuous data was checked by the Kolmogorov-Smirnov test. For univariate comparison of variables being not normally distributed, we used the Mann-Whitney *U*-test for continuous and the χ^2^-test for categorical data. A logistic regression model was used to assess the influence of certain parameters on neurological outcome. In multivariate analysis, we adjusted for clinically relevant covariates.

The biomarkers NSE, SN and tau protein and their interaction terms with the variable sex were added separately to the multivariable logistic regression model in order to assess sex-related interactions of biomarkers predicting neurologic outcome (p_i_ = p_*interaction*_).

Values with a *p*-value < 0.05 were considered as statistically significant. We used IBM SPSS Statistics for Windows, Version 27.0. (IBM Corp, Armonk, NY, USA) to analyze data.

## Results

Of the patients 26% (35/134) were female. Women were significantly older than men (73 years, IQR 56–79 years vs. 62 years, IQR 53–70 years; *p* = 0.038). Furthermore, women tended to receive bystander-initiated CPR more often, to exhibit a shorter time to ROSC and more often a non-shockable rhythm but differences did not reach statistical significance. On ICU admission severity of illness scores (SOFA and APACHE II) were significantly lower in women compared to men, but there was no significant difference in lactate levels (men: 54 mg/dl, IQR 31–96 mg/dl vs. women: 42 mg/dl IQR 31–76 mg/dl; *p* = 0.435). (Table 1).

**Table 1:** Patient characteristics and outcome in female and male patients

	Female (*n* = 35)	Male (*n* = 99)	*p*-value
Age in years, median (IQR)	73 (56–79)	62 (53–70)	0.038
Bystander-initiated CPR, *n* (%)	28 (80)	63 (64)	0.075
Time to ROSC > 20 min, *n* (%)	14 (40)	57 (58)	0.064
Cardiac arrest in-hospital, *n* (%)	6 (17)	11 (11)	0.357
Shockable first monitored rhythm (VFib+VTac), *n* (%)	18 (51)	61 (62)	0.546
SOFA score on admission, median (IQR)	9 (8–10)	10 (9–12)	0.001
APACHE II on admission, median (IQR)	23 (21–26)	26 (22–30)	0.049
ICU-LOS in days, median (IQR)	6 (3–8)	7 (3–12)	0.244
Survival at hospital discharge, *n* (%)	20 (57)	50 (51)	0.499
Standardized mortality ratio (SMR), median (IQR)	1.69 (1.27–1.92)	1.36 (1.22–1.62)	0.072
Poor neurological outcome at hospital discharge, *n* (%)	16 (46)	53 (54)	0.426

*CPR* cardiopulmonary resuscitation, *ROSC* return of spontaneous circulation, *VFib* ventricular fibrillation, *VTAc* ventricular tachycardia, *SOFA* Sequential Organ Failure Assessment, *APACHE II* Acute Physiology and Chronic Health Evaluation, *ICU* intensive care unit, *LOS* length of stay

### Targeted temperature management

Females tended to be treated less often with MTH compared to male patients (11 (31%) vs. 48 (48%); *p* = 0.081) (Table 2a, Fig. 1). There was no difference in duration of hypothermia including cooling down, time on target temperature and rewarming between the sexes (39 h, IQR 36–41 h vs. 39 h, IQR 37–41 h; *p* = 0.858).

**Table 2:** Treatment and complications in female and male patients

*a*	*Female (n* *=* *35)*	*Male (n* *=* *99)*	*p‑value*
Pneumonia, *n* (%)	2 (6)	29 (29)	0.004
MTH, *n* (%)	11 (31)	48 (48)	0.081
WLST, *n* (%)	13 (37)	44 (44)	0.453
Catecholamines on admission, *n* (%)	26 (79)	74 (80)	0.924
*b*	*Female (n* *=* *33*^*a*^*)*	*Male (n* *=* *93*^*a*^*)*	*p‑value*
AKI, *n* (%)	15 (45)	58 (62)	0.091
AKI (stage 3), *n* (%)	5 (15)	24 (26)	0.212
RRT, *n* (%)	3 (9)	17 (18)	0.177

*AKI* acute kidney injury, *RRT* renal replacement therapy, *MTH* mild therapeutic hypothermia, *WLST* withdrawal of life sustaining therapies

^a^8 patients were excluded from AKI staging due to chronic renal insufficiency (> CKD G3)

### Complications

**Fig. 2:**
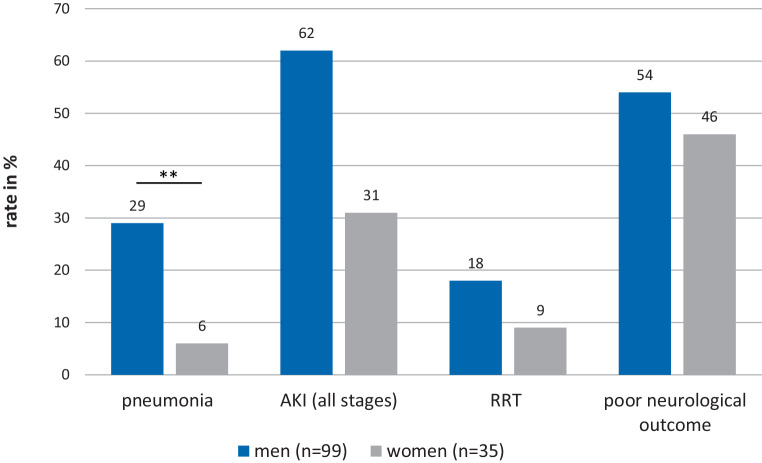
Complications and neurological outcome in men and women after cardiac arrest. *AKI* acute kidney injury—8 patients were excluded from AKI staging due to chronic renal insufficiency (> CKD G3), *n* female = 33, *n* male = 93, *RRT* renal replacement therapy, **p < 0.01)

In male patients (compared to female) we could observe a significantly higher rate of pneumonia (29 (29%) vs. 2 (6%); *p* = 0.004). The percentage of patients receiving catecholamines was almost equally distributed between men and women (Table 2a, Fig. 2).

The rate of AKI was determined in 126 patients after exclusion of 8 patients with CKD > grade 3. Men showed a trend towards higher rates of AKI (all stages), including severe forms of AKI (AKI stage 3) or requirement of RRT. (Table 2b, Fig. 2).

### Withdrawal of life-sustaining therapies

Of 35 female patients, 2 received withdrawal of therapy and 11 withholding of therapy, whereas of 99 male patients, 8 received withdrawal of therapy and 36 withholding of therapy. The percentage of patients with WLST was not significantly different between men and women (Table 2A).

### Neurological outcome, survival and length of stay

Female and male patients showed equal neurological outcome, survival, SMR and LOS on ICU (Table 1, Fig. 2). The median follow-up time was 20 (IQR 11–38) days for patients with good and 4 (IQR 2–9) days with poor neurological outcome.

Female sex was not significantly associated with poor neurological outcome in univariable and multivariable analyses (*p* = 0.613 and *p* = 0.524, respectively); however, we found a significant association between age, time to ROSC > 20 min and the presence of a non-shockable rhythm (Table 3). Rates of poor neurological outcome in women < 50 years were not significantly different to women ≥ 50 years (2/7 (29%) vs. 14/28 (50%); *p* = 0.406).

**Table 3:** Multivariable logistic regression analysis with neurological outcome as dependent variable

	Wald χ^2^ (95% CI)	*p*-value
Age	1.036 (1.004–1.068)	0.026
Sex	0.711 (0.249–2.031)	0.524
MTH	0.713 (0.282–1.806)	0.476
Time to ROSC > 20 min	3.624 (1.501–8.751)	0.004
Shockable first monitored rhythm (VFib+VTac)	0.238 (0.090–0.624)	0.004
SOFA score on admission	1.087 (0.919–1.285)	0.330

*MTH* mild therapeutic hypothermia, *ROSC* return of spontaneous circulation, *VFib* ventricular fibrillation, *VTac* ventricular tachycardia, *SOFA* Sequential Organ Failure Assessment, *CI *confidence interval

### Biomarkers of neurological outcome

We found no interaction between female sex and prognostication of neurological outcome at any time interval within the first 72 h after CPR. For NSE the p_i_ at 24 h was 0.524, at 48 h 0.221 and at 72 h 0.519. SN had a *p*_i_ of 0.893, 0.573 and 0.545 and tau protein a *p*_i_ of 0.270, 0.635 and 0.110 at 24, 48 and 72 h, respectively. Diagnostic accuracy of NSE, SN and tau protein at 0–24 h, 24–48 h and 48–72 h to predict poor neurological outcome is presented in Table 4.

**Table 4:** Receiver operating characteristics (ROC) analysis for the prediction of poor neurological outcome by several biomarkers

AUC [95% CI]	0–24 h	24–48 h	48–72 h
Tau	0.645 [0.540–0.750]	0.775 [0.679–0.870]	0.783 [0.671–0.894]
SN	0.708 [0.611–0.806]	0.711 [0.606–0.816]	0.552 [0.417–0.687]
NSE	0.860 [0.778–0.943]	0.883 [0.787–0.979]	0.881 [0.815–0.946]

*ROC* receiver operating characteritic, *AUC* area under the curve, *Tau* tau protein, *SN* secretoneurin, *NSE* neuron-specific enolase, *CI* confidence interval

## Discussion

In our study investigating sex differences in post-CA management and outcome we found the following characteristics: the proportion of female patients was low. Although they were significantly older, they presented with significantly lower severity of illness scores (SOFA and APACHE II) on admission. Typical complications during the post-CA period like pneumonia and AKI were observed less frequently in women. Despite that, both neurological outcomes and hospital survival were equal between sexes.

The proportion of female patients admitted to our ICU was 26%, which is within the reported range of 20–28% in similar studies [3, 4, 16, 17]. Therefore, our study reflects the typical sex distribution of a CA cohort. We do not know the number of female OHCA patients not admitted to ICU. In larger studies the proportion of females with OHCA ranged from 20% [39] to 36% [40]. In a previous meta-analysis, no significant difference in survival from OHCA to hospital admission between females and males could be established [2].

Women seem to be less often affected by CA, but present with more unfavorable pre-CA profiles, such as higher age [11], lower rates of bystander CPR [24] and less shockable rhythms [10, 11, 17]. Contrarily, they appear to have higher rates of ROSC after OHCA [7, 10, 11, 20]. In our study, women despite being significantly older showed a trend towards higher rates of bystander-initiated CPR and lower rates of prolonged CPR. This may have resulted in significantly lower SOFA and APACHE II scores at admission. A recent study investigating why women might receive less bystander CPR, revealed that members of the general public often perceive fears about inappropriate touching, accusations of sexual assault or fear of causing injury [41]. The opposite trend observed in our study could be influenced by the fact that several patients had an in-hospital CA (*n* = 17, 13%, 11 men and 6 women) and received bystander CPR from trained medical personnel. Furthermore, there might be regional and cultural differences as well as different levels of education in provision of CPR.

Concerning post-CA management, it has been reported that women are less often treated with MTH than men [3, 12–15]. Apart from the finding that MTH may be underutilized in women [3, 12–15], it was further observed that women less likely benefit from MTH [42] and neurological outcomes were poorer in women despite equal treatment with targeted temperature management. No interaction between sex and the effect of targeting 33° vs. 36 °C could be found [43]. In our study, rates of MTH treatment in men and women were not significantly different. Moreover, there was no significant association of sex or MTH with neurological outcome in multivariate logistic regression analysis.

In our cohort, male patients more likely developed complications on ICU. Men showed a significantly higher rate of pneumonia compared to women, which is in accordance with a similar study including comatose resuscitated patients after OHCA treated with MTH [17]. This potentially higher susceptibility of men to pneumonia, is supported by recent data from a mouse model suggesting that females might be protected from infections through estrogen-driven antibodies [44].

Reported rates of AKI frequently complicating CA range between 12% and 40% [45–47]. In a large cohort trial of hospitalized patients, males have shown to develop AKI requiring dialysis 2.2 times more likely than females [18]. Development of AKI after CA as a result of ischemia/reperfusion injury (IRI) is driven by a systemic inflammatory response syndrome following ROSC leading to further tissue damage [48–50]. Based on experimental data female sex seems to play a kidney-protective role in AKI mediated by effects of sex hormones. In models of ischemic AKI after IRI, females showed less functional renal impairment and tissue damage. Furthermore, there are differences in the inflammatory, hemodynamic and humoral responses to IRI [51–53].

In our study, we could observe only a trend to higher rates of AKI in male patients. Concerning post-resuscitation shock, as a possible reason for development of AKI, men and women seem to be equally affected, as the lactate levels on admission and the use of catecholamines were not significantly different.

Overall, adverse events in the post-resuscitation period are more often occurring in men, which should be taken into account in daily practice.

The frequency of therapy limitations such as withdrawing/withholding of therapy (43%) was similar to a previous study identifying factors associated with WLST. It was observed that patients with WLST were more likely to be female [54]; however, we could not find a significant difference between men and women, which is in line with previous observations in a similar cohort [17].

In our study, we found no significant difference in neurological outcome and survival between men and women. Previous studies showed conflicting results regarding survival [9, 10, 19–24] and neurological outcome [17, 23, 25–28]. Similar outcomes for both sexes were found in our study despite lower severity of illness scores at admission in women. We speculate that the significantly higher age of women might be counterbalanced by a lower rate of complications.

Previous reports demonstrated a lower incidence and better outcomes after OHCA for women within an age of 13–49 years compared to men of the same age group [55]. Women of childbearing age (15–44 years) also seem to be more likely to survive to hospital discharge after in-hospital CA compared to men [56]. It is speculated that this might be also attributed to a protective role of female sex hormones in hypoxic brain injury [57–59]. In our cohort, we could not observe a significant difference in neurological outcome between females < 50 years compared to ≥ 50 years; however, our cohort was very small.

For prognostication of poor neurological outcome, we applied a multimodal approach. Medical decision making followed a rigorous protocol. Beside the gold standard serum NSE, SN and Tau protein have proved to be reliable predictors of poor neurological outcome [30, 34]. In none of the biomarkers was the predictive value affected by sex suggesting that biomarkers can be equally applied to men and women.

### Strengths and limitations

In our study, we analyzed for the first time prognostic biomarkers of neurological outcome in combination with pre-cardiac arrest profile, MTH treatment and complications after CA in men and women. Among the limitations of the study are its relatively small sample size resulting in a low number of female patients and its design as a single centre study. Furthermore, this was a retrospective analysis of a prospective study that was initially targeted to determine predictive performance of serum biomarkers for neurological outcome. In this context we had 10 drop-outs because of death not related to hypoxic ischemic encephalopathy as we wanted to avoid misclassification to a neurological death and confounding of outcome assessment. Therefore, results have to be interpreted with caution and confirmed in larger cohorts. Neurological outcome was determined at hospital discharge following a rigorous protocol defining withdrawal of therapy [30]. Although the median follow-up time for patients with good neurological outcome was relatively long, we cannot rule out later improvement or worsening of neurological outcome; however, CPC at hospital discharge has shown to be a good predictor of long-term outcome [60].

## Conclusion

The proportion of female patients in our study was low and women were significantly older suggesting a worse pre-arrest profile. During the post cardiac arrest-period, men and women seemed to receive equal treatment with mild therapeutic hypothermia and there was no significant difference in the frequency of withdrawal of life-sustaining therapies. Women developed fewer complications reflected by a significantly lower rate of pneumonia and a trend to lower rates of acute kidney injury; however, female sex was not associated with better neurological outcome after cardiac arrest. Importantly, the performance of biomarkers to predict poor neurological outcome was not affected by sex.

## Supplementary Information

Categories of the Cerebral Performance Categories (CPC) Scale and Medical Decision Making
