# Supplementary material for: Postresuscitation care and prognostication after cardiac arrest—Does sex matter?
Source: Wien Klin Wochenschr. 2022 Apr 5;134(17-18):617–25. doi: 10.1007/s00508-022-02026-x (PMC9489581; doi:10.1007/s00508-022-02026-x)
Supplement: Supplementary file 1 — Categories of the Cerebral Performance Categories (CPC) Scale and Medical Decision Making [file 508_2022_2026_MOESM1_ESM.docx]

**Detailed description of the 5 categories of the Cerebral Performance Categories Scale (CPC)** *(Edgren E, Hedstrand U, Kelsey S, Sutton-Tyrrell K, Safar P (1994) Assessment of neurological prognosis in comatose survivors of cardiac arrest. BRCT I Study Group. Lancet 343:1055-1059.)***:**

CPC 1: Good cerebral performance (normal life: Conscious, alert, able to work and lead a normal life; may have minor psychological or neurologic deficits (mild dysphasia, non-incapacitating hemiparesis, or minor cranial nerve abnormalities).

CPC 2: Moderate cerebral disability (disabled but independent): Conscious, sufficient cerebral function for part-time work in sheltered environment or independent activities of daily life (dress, travel by public transportation, food preparation); may have hemiplegia, seizures, ataxia, dysarthria, dysphasia, or permanent memory or mental changes.

CPC 3: Severe cerebral disability (conscious but disabled and dependent): Conscious, dependent on others for daily support (in an institution or at home with exceptional family effort); has at least limited cognition. This category includes a wide range of cerebral abnormalities, from patients who are ambulatory but have severe memory disturbances or dementia precluding independent existence to those who are paralyzed and can communicate only with their eyes, as in the locked-in syndrome.

CPC 4: Coma/vegetative state (unconscious): Unconscious, unaware of surroundings, no cognition. No verbal or psychological interaction with environment.

CPC 5: Brain death (certified brain dead or dead by traditional criteria).

**Medical decision making**

Decisions about further medical treatment in terms of withdrawal or withholding of therapy were taken after completion of MTH, complete weaning off sedation and diagnostic procedures in an interdisciplinary conference of treating intensivists and neurologists. The decision was based on clinical, neurophysiological and imaging data as well as concomitant underlying diseases of the patient. NSE levels were available for the prognostication process. However, no decision was based only on this biomarker. The number of patients belonging to following subgroups was documented: (1) Withdrawal of therapy, (2) Withholding of therapy.

Findings allowing withdrawal (1) or withholding (2) of therapy are listed below:

Brain death due to cerebral herniation, early severe myoclonus status and absence of bilateral median nerve somatosensory evoked potentials (SSEP) allowed discontinuation of active treatment.

Withdrawal and withholding of therapy could also be done if patients stayed in persistent coma with negative SSEP or refractory status epilepticus after completion of MTH, complete weaning off sedation and diagnostic procedures.

Further, limiting of life- sustaining treatment could be considered in patients who retained SSEP, but did not improve in GCS for longer than 72 hours after weaning off sedation.

Importantly, those patients had to be re- evaluated clinically and by means of EEG and neuroimaging.

Finally maximal therapy could be stopped due to ethical reasons (e.g. based on underlying diseases).

The medical consensus was discussed with the patient´s family also considering the patient´s presumed will. Secretoneurin and tau protein levels were not available for the treating physicians and did not influence this critical process in any case.
